# Acute Physiological Responses to High-Intensity Resistance Circuit Training vs. Traditional Strength Training in Soccer Players

**DOI:** 10.3390/biology9110383

**Published:** 2020-11-07

**Authors:** Cristian Marín-Pagán, Anthony J. Blazevich, Linda H. Chung, Salvador Romero-Arenas, Tomás T. Freitas, Pedro E. Alcaraz

**Affiliations:** 1 Research Center for High Performance Sport, Catholic University of Murcia, 30107 Murcia, Spain; lhchung@ucam.edu (L.H.C.); tfreitas@ucam.edu (T.T.F.); palcaraz@ucam.edu (P.E.A.); 2 Centre for Exercise and Sports Science Research, School of Medical and Health Sciences, Edith Cowan University, 6027 Joondalup, Australia; a.blazevich@ecu.edu.au; 3 Faculty of Sport Science, Catholic University of Murcia, 30107 Murcia, Spain; sromero@ucam.edu; 4 NAR-Nucleus of High Performance in Sport, São Paulo 04753060, Brazil

**Keywords:** aerobic fitness, muscle strength, oxygen uptake, heart rate, football

## Abstract

**Simple Summary:**

Strength training is a key factor for soccer players, but at amateur levels, it is difficult to apply due to the lack of infrastructure and short training time. In this regard, high-intensity resistance circuit-based training could be a suitable method to solve these issues. Circuit training can improve the cardiorespiratory and metabolic responses while reducing training time by 66%. The effects of circuit training could contribute to improving aerobic fitness and body composition in soccer players.

**Abstract:**

The aim of this study was to evaluate and compare the cardiorespiratory and metabolic responses induced by high-intensity resistance circuit-based (HRC) and traditional strength (TS) training protocols. Ten amateur soccer players reported to the laboratory on four occasions: (1) protocol familiarization and load determination; (2) maximal oxygen consumption test; (3) and (4) resistance training protocols (HRC and TS), completed in a cross-over randomized order. In both protocols, the same structure was used (two blocks of 3 sets × 3 exercises, separated by a 5-min rest), with only the time between consecutive exercises differing: TS (3 min) and HRC (~35 s, allowing 3 min of local recovery). To test for between-protocol differences, paired t-tests were applied. Results showed that oxygen consumption and heart rate during HRC were 75% and 39% higher than TS, respectively (*p* < 0.001). After the training sessions, blood lactate concentration at 1.5, 5 and 7 min and excess post-exercise oxygen consumption were higher in HRC. The respiratory exchange ratio was 6.7% greater during HRC, with no between-group differences found post-exercise. The energy cost of HRC was ~66% higher than TS. In conclusion, HRC training induces greater cardiorespiratory and metabolic responses in soccer players and thus may be a time-effective training strategy.

## 1. Introduction

Soccer is a sport requiring intermittent bouts of exercise, alternating short periods of high intensity activity with long periods of low intensity [1]. Power, velocity, and agility are fundamental aspects of a soccer player’s performance because they are the basis for performing different actions such as high-velocity and short-duration movements (1–7 s), jumps, and changes of direction. These requirements highlight the need for implementing training schemes specifically aimed at developing maximal force production capacity (e.g., strength, power) [2,3]. In fact, maximal strength development is considered fundamental in team sports as it is the basis for power production [4] and short-distance sprint performance [5,6]. Additionally, strong evidence has shown that team sport athletes with higher levels of strength have a considerably lower injury incidence [7,8] and are able to better tolerate larger week-to-week changes in training load [9].

Several authors [10,11] have recommended low-volume high-intensity resistance training in this population, utilizing loads of ~65–90% of the one-repetition maximum (RM) [10,12]. In particular, traditional strength training, with loads of around 85–90% of 1-RM (~6-RM) and long inter-set rest periods (2–5 min), has been used to achieve increases in maximal strength [13,14], muscle mass [15], and work economy [16]. Therefore, resistance exercise intensity seems to be a key variable to consider in order to improve strength and athletic performance [17,18]. 

In soccer, a challenge often found with the planning of players’ physical development programs is that they must focus on the concurrent development of several physical parameters (power, velocity, acceleration ability, etc.). That is, they must consider not only muscular strength development but also aerobic (and anaerobic) capacity [19,20,21]. However, during traditional strength training, heart rate (HR) tends to remain relatively low (around 60% of maximum) [22], and the stimulus is likely to be inadequate to generate significant cardiorespiratory adaptations [23]. 

With this in mind, resistance circuit-based training has been shown to be an effective method to improve strength and endurance concurrently in the same session [24]. Particularly, in trained individuals, high-intensity resistance circuit-based training (HRC; a method in which high loads are utilized with short inter-set rests) may be a suitable alternative to achieve maximal strength improvements similar to traditional strength training [15], but with a significantly greater cardiovascular response [22]. In essence, HRC training combines the advantages of traditional strength training (TS) (i.e., high loads to generate muscular adaptations) [15] and traditional light-load circuit resistance training (for stimulation of cardiorespiratory parameters) [25]. Further, HRC training has been shown to decrease total training time by around two-thirds (by 66%) [22] and to significantly reduce fat mass after 8 weeks of intervention in trained men [15]. Thus, some authors have speculated that HRC might be an appropriate resistance training method for athletes because it may allow for improvements in maximal strength, anaerobic and aerobic endurance, and body composition in a time-efficient manner [26]. 

Nevertheless, research on the acute effects of HRC is scarce, and the investigations of this training methodology have focused on identifying the fatigue-induced responses after a single bout of HRC in recreationally active males [27,28] or basketball players [29]. To our knowledge, no studies have examined the acute effects of HRC on aerobic and anaerobic metabolism or cardiorespiratory responses in amateur soccer players. In addition, no evidence exists pertaining to the energy cost (EC) or excess post-exercise oxygen consumption (EPOC) during and immediately after an HRC session. 

Therefore, this study aimed to investigate the acute physiological responses to HRC training in amateur soccer players by documenting and comparing both cardiorespiratory and metabolic responses induced by HRC and TS. Based on previous results, we hypothesized that HRC would induce a greater response from the cardiorespiratory system and, consequently, a higher metabolic stress.

## 2. Method

### 2.1. Participants

Ten amateur soccer players volunteered to participate in the present study (Table 1). Only field players with a minimum experience of 3 years in amateur soccer competition were recruited. All participants were adults (19–30 years old) and were informed of the study procedures before signing an informed consent document. Players reported that they did not take ergogenic aids or medications that might influence performance, and only participants without musculoskeletal injuries in the previous 6 months or cardiorespiratory disorders that required health professional interventions were included in the study. The study was conducted according to the Helsinki Declaration (1964; revised in 2014) and the experimental protocol was approved on 10 March of 2017 by the Catholic University of Murcia Ethics Committee (code: CE031704).

### 2.2. Experimental Design

A randomized, counterbalanced, crossover study design with familiarization was used. The cardiorespiratory and metabolic variables were HR, oxygen consumption during exercise ($\dot{V}$O_2_), EPOC, post-exercise blood lactate concentration ([La^−^]), average respiratory exchange ratio (RER), and total energy cost (EC).

**Figure 1:**
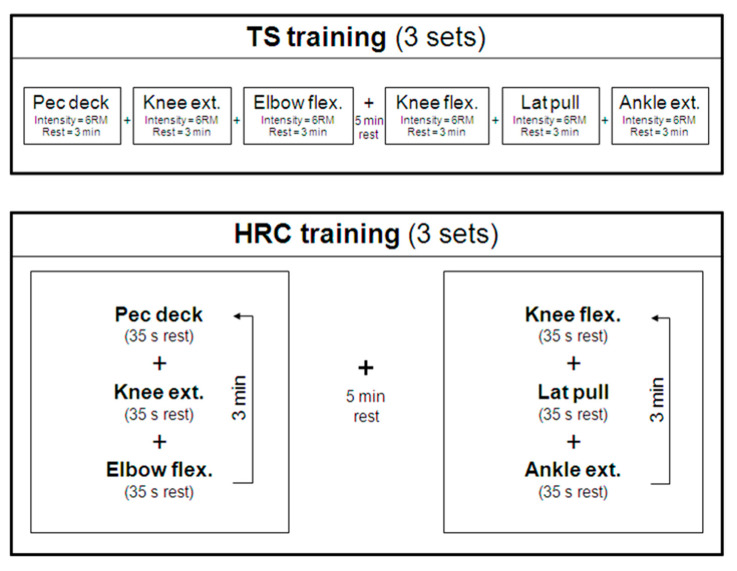
Experimental design. HRC = high-intensity resistance circuit-based training; TS = traditional strength training; ext. = extension; flex. = flexion. See text for more details.

Participants visited the laboratory on four occasions, with 72 h separating each visit. On day 1, participants performed a familiarization session of all tests and training exercises. Additionally, their 6-repetition maximum (6-RM) loads were determined for the pec deck, knee extension, elbow flexion, knee flexion, lat pulldown, and ankle extension exercises, according to standard procedures of American College of Sports Medicine (ACSM) in 2002 [23]. On day 2, an incremental treadmill test was performed, and, on the subsequent two visits, participants completed one of the two resistance training programs in a randomized but counterbalanced order (Figure 1). Oxygen (O_2_) utilization and EC were continuously measured during the training sessions and during the first 20 min of recovery. In all tests, participants were assessed by the same investigator, using the same protocol and at the same time of day. In the 24 h before each session, volunteers were required to (a) avoid the ingestion of caffeine or other metabolism-altering supplements and drugs, (b) engage in no physical activity, (c) maintain themselves well hydrated and not change their habitual diet, and (d) avoid strenuous non-exercise related efforts.

### 2.3. Procedures

Treadmill oxygen consumption assessment. In the second visit, an incremental treadmill exercise test was used to assess aerobic capacity. After a 2-min warm-up walking at 4 km·h^−1^ at 1% inclination, the treadmill velocity was increased to 7 km·h^−1^ and then increased 1 km·h^−1^ at each 1-min stage until exhaustion. During the exercise test, participants breathed through a face mask, which allowed breath-by-breath analysis of O_2_ and carbon dioxide (CO_2_) using a portable gas-analysis system (Oxycon Mobile, Jaeger-Viasys^TM^, Hoechberg, Germany). Oxygen consumption was measured by comparing input to output data to determine what volume was used. Ventilation ($\dot{V}$E), $\dot{V}$O_2_, and $\dot{V}$CO_2_ were determined as a time-average of the breath by breath report. Before each testing day, the O_2_ and CO_2_ analysis system was calibrated following the manufacturer guidelines. HR was monitored and continuously recorded (Polar T61, Kempele, Finland). For data analysis, $\dot{V}$O_2_, $\dot{V}$E, $\dot{V}$CO_2_, and HR values were averaged every 20 s. The test was considered maximal when at least two of the following criteria were met: (a) RER > 1.15; (b) a plateau in $\dot{V}$O_2_ was obtained despite an increase in workload (increase, 2.0 mL·kg^−1^·min^−1^ between the last two loads); and (c) maximum volitional exhaustion. Peak $\dot{V}$O_2_ was defined as the mean $\dot{V}$O_2_ during the last minute of the exercise test. The second ventilatory threshold (VT_2_) was determined by two experienced, independent physiologists using the ventilatory equivalents method [30].

Resistance training oxygen consumption. Participants performed one of the two resistance training protocols in a counterbalanced design. The second exercise protocol was performed 72 h after the first session. $\dot{V}$O_2_ normalized to body mass ($\dot{V}$O_2_/BM) was measured during exercise and rest intervals and then averaged to be expressed as mlO_2_∙kg^−^^1^∙min^−^^1^. The total $\dot{V}$O_2_ was also calculated for both conditions. The total $\dot{V}$O_2_ for a given bout was defined as the overall $\dot{V}$O_2_ during exercise and the 5-min recovery period between blocks. Moreover, before each training session, $\dot{V}$O_2_ was measured for 5 min with the participants seated, to determine the resting values of $\dot{V}$O_2_ and HR. The values used for analysis corresponded to the averages of the last 3 min in that procedure. 

Resistance training energy cost. EC was calculated using the equation described by Weir [31]. 

Post-resistance training evaluation. Immediately following each training session, participants sat on a chair for 20 min with the gas analyzer recording. The mean values of HR, RER, EPOC, and post-exercise EC were measured continuously during the abovementioned period. The EPOC was calculated as (1):

(1) $$EPOC=\dot{V}O_{2}\;-\;rest\;\dot{V}O_{2}$$

Blood lactate concentration. Resting [La^-^] was determined from a blood drop obtained from the left earlobe, with the participant in a seated position following a 1.5 min resting period after the warm-up. Post-workout [La^-^] was also measured with the participants in a seated position at 1.5, 5, and 7 min after completing the workout. Calibration of the lactate testing device (Lactate Pro, Arkray, Kyoto, Japan) was performed prior to use, according to the procedures outlined by the manufacturer. After sterilizing the left earlobe, a puncture was made with a sterile lancet. The first drop of blood was wiped away. The second drop of blood was applied to an assay strip and inserted into the lactate testing device.

### 2.4. Resistance Training Sessions

A 5-min general warm-up [5] that included jogging on a treadmill and a 5-min active stretching routine of all major muscle groups was performed before the workout. In addition, a specific warm-up was completed. It consisted of 3 sets of 3 exercises (pec deck, knee extension, and elbow flexion) performed according to the following sequence: 10 repetitions at 50% of 6-RM of each exercise; 1-min rest; 8 repetitions at 75% of 6-RM; 2-min rest; and repetitions to failure with the 6-RM load. The 6-RM load was adjusted by approximately ±2.5% if a participant performed ±1 repetitions and was adjusted by approximately ±5% if a participant performed ±2 repetitions [23]. Participants rested for 3-min before starting the workout. During this period, the gas analysis mask and equipment were properly placed on the participant after they were positioned to perform the first exercise. A facemask that covered the participant’s mouth and nose was attached to a bidirectional digital flow valve and fastened with the use of a mesh hairnet and Velcro^©^ straps (Knutsford, United Kingdom). The specific warm-up sequence was recorded and performed on each session. During the training session, training supervisors motivated participants equally to complete the maximum number of repetitions possible in each set.

Traditional strength training session (TS). Three sets of 6 exercises, divided into 2 blocks, were performed: block 1-pec deck, knee extension, elbow flexion (preacher curl); and block 2-knee flexion (leg curl), lat pulldown, ankle extension (seated calf raise). The exercises were chosen to emphasize both major and minor muscle groups using single- as well multi-joint exercises, based on the recommendations of the American College of Sports Medicine (ACSM) [32]. In every session, the participants lifted loads that allowed only 6 repetitions to be performed (6-RM, ~85–90% of 1-RM), with a 3-min rest between sets and 5-min rest between blocks. The 3 sets of 3 exercises in block 1 were completed before block 2 (Figure 1). The eccentric phase of each exercise was performed in 3 s (controlled by digital metronome), whereas the concentric phase was performed at maximum velocity. The participants were supervised by an experienced strength and conditioning specialist to ensure that volitional fatigue was achieved safely and to strictly control resting periods.

High-intensity resistance circuit-based session (HRC). The HRC session differed from the TS session only in the rest interval between consecutive exercises. Training in the HRC session was completed in two short circuits (blocks), with a 35-s rest between exercises (which allowed enough time to move safely from one exercise to the next), a 3-min rest between each series of 3 exercises within a block, and a 5-min rest between the blocks. Each block was performed three times. The first and second blocks in the HRC program included the same exercises as in TS (Figure 1) as well as the same warm-up, exercise intensity, and volume. Again, the participants were supervised by an experienced strength and conditioning specialist.

### 2.5. Statistical Analysis

SPSS statistical software v19.0 (IBM Company, New York, NY, USA) for Windows was used to analyze all data. Participants’ physical characteristics are reported as means and standard deviation (SD). Normal distribution and homogeneity of data were checked with Kolmogorov–Smirnov and Levene tests, respectively. To determine differences between protocols, a paired t-test was applied, except for the EPOC, in which an ANOVA of repeated measures was used with a Bonferroni post-hoc. Statistical significance was set at *p* < 0.001 for all analyses. Effect sizes (ES) were calculated using Cohen’s equations [33]. Threshold values for ES statistics were: >0.2, small; >0.6, moderate; >1.2, large; >2.0, very large; and >4.0, nearly perfect [34].

## 3. Results

For clarity, the cardiorespiratory and metabolic effects of each training protocol are shown separately for: (i) during the training session, and (ii) post-training session.

### 3.1. During the Training Session

Paired t-test was used to detect between-condition differences in baseline values. Significantly higher values (*p* < 0.001) were found for all variables measured during HRC than TS (Table 2).

**Figure 2:**
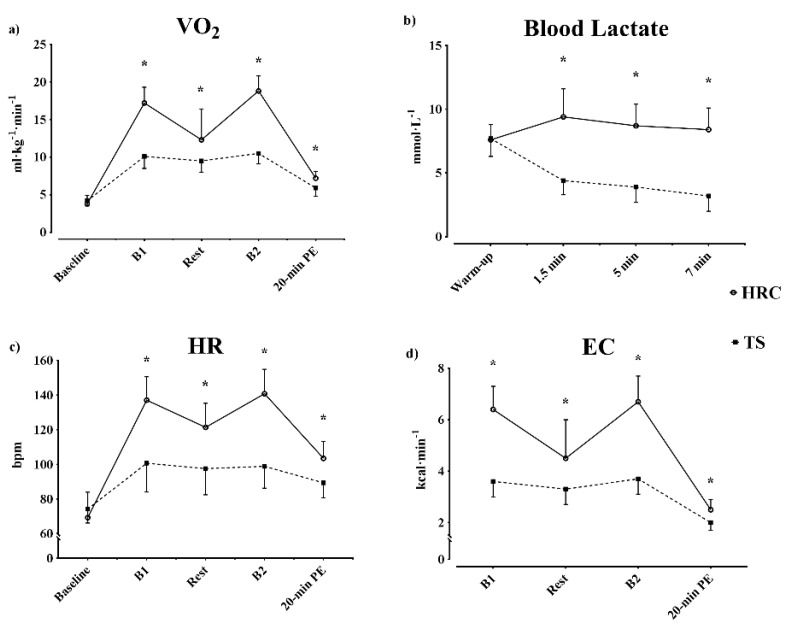
Oxygen consumption, blood lactate, heart rate, and energy cost, during HRC training or TS training and mean in the final rest period. (**a**); oxygen consumption; (**b**); blood lactate concentration; (**c**); heart rate; (**d**); energy cost; HRC = high-intensity resistance circuit-based training; TS = traditional strength training; VO_2_/BM = relative oxygen consumption; HR = heart rate; B1 = block 1; B2 = block 2; average = mean of B1 and B2. * = significant differences from HRC, *p* < 0.001.

$\dot{V}$O_2_/BM and $\dot{V}$O_2_ at VT_2_ ($\dot{V}$O_2,VT2_) were significantly higher (*p* < 0.001) in HRC. Both respiratory variables responded similarly, corresponding to an aerobic demand ~75% greater during HRC than TS. The average HR was also significantly elevated (*p* = 0.001) in HRC, with a value ~39% greater than in TS. Additionally, the respiratory exchange ratio in HRC was ~6.7% higher than in TS (*p* < 0.001). Finally, HRC presented a ~66% greater EC when compared to TS (*p* = 0.001), as seen in Figure 2d.

### 3.2. Post-Training Session

Figure 2 shows the changes in [La^-^] after each training protocol, as well as changes in $\dot{V}$O_2_, HR, and RER during the training sessions. Following HRC, [La^−^] was higher at all time points (1.5 min = 9.4 ± 2.2; 5 min = 8.7 ± 1.7; 7 min = 8.4 ± 1.7) than in TS (1.5 min = 4.4 ± 1.1; 5 min = 3.9 ± 1.2; 7 min = 3.2 ± 1.2 mmol·L^−1^), as shown in Figure 2b (*p* < 0.001; ES = 2.75; 3.12; 3.38, respectively). Considering the average of the three measurements, [La^-^] was 133% higher in HRC compared to TS. 

**Figure 3:**
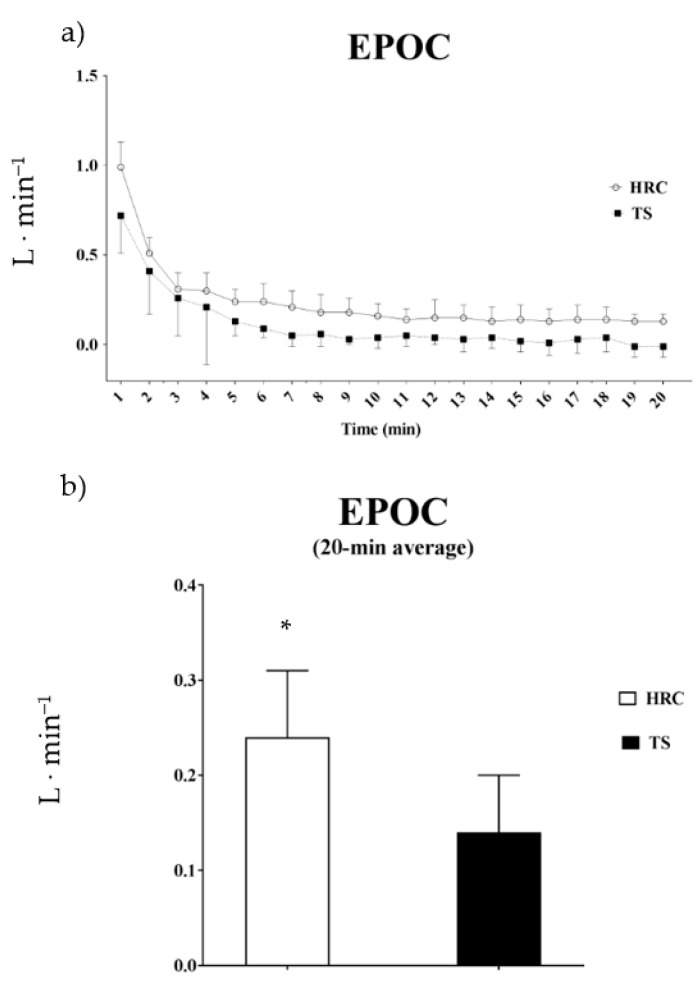
Excess post-exercise oxygen consumption after training session. EPOC = excess post-exercise oxygen consumption after training session. (**a**); excess post-exercise oxygen consumption per minute; (**b**); average of 20-min of excess post-exercise oxygen consumption; HRC = high-intensity resistance circuit-based training; TS = traditional strength training; * = significant differences, *p* ≤ 0.001.

During the recovery period (Table 3), EC was significantly greater after HRC (*p* < 0.001). Moreover, in the 20 min following the training, an increased EPOC was observed in HRC (126% greater than TS), as shown in Figure 3. The mean HR was also higher in HRC (*p* < 0.001). After the session, no differences between protocols were found for RER.

## 4. Discussion

The main purpose of the present study was to quantify and compare the acute physiological effects of a high-resistance circuit-based (HRC) and a traditional strength (TS) training session in a sample of amateur soccer players. The most interesting finding was that the cardiorespiratory and metabolic responses were significantly higher during the HRC when compared to TS, despite the same loads (i.e., 6-RM) being lifted during the sessions and a much shorter required training time (–66%). Of note, the present results are in accordance with the proposed hypothesis and with previous research in recreationally active male participants [22].

An important discovery was that the $\dot{V}$O_2_ responses during the training sessions were markedly different between protocols, with greater values found in the HRC condition. These results suggest that HRC training may simultaneously target cardiovascular and strength adaptations. In line with this, a recent meta-analysis concluded that resistance circuit-based training, independent of the protocol used, may be an effective strategy to increase $\dot{V}$O_2max_ in sedentary, active, and trained individuals [24]. Nevertheless, further research is necessary to confirm this assumption given that the $\dot{V}$O_2_ values obtained during HRC were slightly lower than those recommended by the ACSM (≥40% of $\dot{V}$O_2max_) for improving aerobic capacity [35]. 

As previously stated, during a soccer match, players work in the aerobic zone for ~98% of the time [36], which explains why achieving improvements in $\dot{V}$O_2max_ is considered a major objective in this sport. In fact, soccer players with higher $\dot{V}$O_2max_ have been found to recover faster [37] and cover greater distances during a match [38,39,40]. A major consideration for coaches and athletes, however, is that soccer players can only devote a limited time to strength and conditioning practices, so finding ways to develop both strength and cardiovascular fitness qualities simultaneously may have substantial benefits. In this context, HRC training may be a useful and time-efficient tool for improving aerobic fitness (while in the weight room), considering that the duration of a HRC session is 66% less than a TS bout [22].

Additionally, worth noting is the fact that significant differences were observed in the exercise intensity relative to VT_2_, expressed as a percentage. The time spent above VT_2_ was ~82% greater in HRC than TS, as the soccer players trained at ~38% and ~22% of $\dot{V}$O_2,VT2_ in HRC and TS, respectively (Table 2). Unfortunately, there are no similar studies in which the exercise intensity relative to VT_2_ was evaluated, so it is not possible to compare our data with others presented in the literature. Importantly, during a standard soccer match (90 min), players work close to the anaerobic threshold [41]; thus, training at or above this threshold could be considered an important preparation strategy. Along these lines, future studies should examine the long-term cardiovascular and VT_2_ adaptations in athletic populations.

The present results also indicate greater involvement of the cardiovascular system during HRC training, as can be observed by the higher HR responses in this protocol when compared with TS training. The noteworthy aspect herein is that the greater cardiovascular load in HRC was imposed despite the training intensity and volume being the same in both protocols (3 sets × 6 exercises × maximum repetitions at 6-RM intensity). This would likely be due to the circuit configuration of the training potentially stimulating adaptations that improve cardiovascular function and, ultimately, endurance, since inter-set recovery during resistance training has been previously shown to have an impact on cardiovascular responses [42]. HR_max_ achieved in HRC was approximately 71% of the maximum, which is well within the 60–90% range suggested by the ACSM [23] for the development of cardiorespiratory fitness and promotion of body composition changes but quite inferior to HR values reported for field-based training approaches (i.e., small-sided games) [43]. This indicates that HRC should not be seen as an alternative to, for example, small-sided games, but as a valuable method to increase the stimulation of the cardiorespiratory system during resistance training in the weight room. Similar results were obtained by Alcaraz et al. [22] in trained men during HRC training (~71% of HR_max_) versus TS training (~62% of HR_max_). The authors concluded that HRC training was associated with a reduced training time yet a greater intensity with respect to the HR response. Other studies have shown that participants worked at similar HR levels during a standard circuit training session [44,45,46,47], although HR responses may be influenced by the exercises used in the training. In accordance with the $\dot{V}$O_2_ data, the HR results indicate that HRC training could lead to cardiorespiratory improvements after a long-term intervention. Still, this hypothesis should be explicitly tested in the future [48].

The metabolic response was also found to be significantly higher during HRC, with greater [La^-^] at all time points when compared to TS training. These data show that the anaerobic metabolism activation was greater during HRC and, consequently, that the anaerobic energy contribution was higher. A previous study [49] revealed slightly higher [La^-^] values (10.5 ± 2.1 mmol·L^−1^) after a 5-min high-intensity interval resistance training session than those obtained after the HRC herein, when 6-RM loads were used by resistance-trained males. These differences could be explained by the dissimilar rest periods allowed between consecutive exercises (HRC = 35 s; HIRT = 20 s) or sets (3 min vs. 2.5 min respectively). Regardless, the present data suggest that HRC training may provide an important stimulus for improvements in anaerobic fitness (e.g., blood lactate tolerance and clearance).

An additional indication that HRC training imposed a notable stress on the anaerobic metabolism is the fact that the RER was significantly higher during this protocol. Again, it appears that there is a greater upregulation of anaerobic metabolism during HRC training. These RER values were higher than those obtained by Beckham et al. [45], who reported values of 1.01 ± 0.11 during a circuit weight training session with moderate loading (10.5 kg). RER is proposed to indicate metabolic predominance, which, together with the increased EC and EPOC [49], could explain some of the changes previously shown in body composition after a HRC training program [15,50]. In fact, previous research has reported a higher energy expenditure following circuit-based resistance training protocols [49].

Traditionally, it has been considered that circuit training with high loads produces a slightly lower aerobic response than circuit weight training with low and moderate loads (≤60% of 1-RM) [13,51]. However, the latest evidence suggests the opposite, given that increases in $\dot{V}$O_2max_ were found to be greater when higher intensities were used in the resistance circuit-based training [24]. Of note, the intensity used in the present study (6-RM = ~85% of 1-RM) corresponds to the ACSM and National Strength and Conditioning Association (NSCA) recommendations for strength development in young trained individuals [23,32,52] and should, thus, provide a considerable strength and hypertrophy stimulus. In fact, high-intensity resistance training has consistently been shown to be effective for eliciting muscular adaptations in healthy adults [15,53]. Since HRC combines characteristics of both training types (traditional circuit training and traditional strength training), it may be a useful tool for improving soccer players’ strength and cardiovascular function while in the weight room, with the upside of being completed in a shorter training time. In addition, from an injury prevention perspective, this method could conceivably lead to increased robustness and decreased risk of injury, given its potential to increase maximal strength levels [8,9].

In summary, HRC training could be a suitable alternative to minimize the time spent in the weight room whilst potentially developing several physical qualities simultaneously (e.g., strength and endurance). However, future studies are required to assess the long-term impact of HRC training in soccer players and other athletes, particularly with the use of more specific and compound exercises. The main limitations of the present study were the small sample size and the fact that the exercises performed in both training protocols did not include movement patterns that mirror those commonly used in soccer. Nevertheless, players in this sample had limited resistance training experience and, as such, the exercises prescribed were appropriate for the participants’ level. Moreover, the characteristics of the devices used limited exercise prescription (players had a portable gas analyzer strapped to the chest which would make it extremely challenging to perform more explosive, closed-chain compound exercises during the training session). An important limitation is that the present results were obtained in an amateur population, so it cannot be assumed that higher-level (e.g., professional) players, who might have different training histories, genetic profiles and a higher aerobic capacity, would respond equally. Acute effects of HRC in these populations should be explicitly examined in future studies.

## 5. Conclusions

HRC elicits greater cardiovascular and metabolic responses than TS training in semi-professional soccer players. Therefore, HRC could be considered a time-efficient and useful method for potentially inducing cardiovascular and strength improvements, optimizing the time spent in the weight room by developing both qualities (i.e., cardiovascular-and strength-related) simultaneously.

## Figures and Tables

**Table 1:** General characteristics of participants (mean ± SD) (*n* = 10).

Age (y)	Height (cm)	Mass (kg)	$\dot{V}$O_2max_ (mL·kg^−1^·min^−1^)	VT_2_ (% of $\dot{V}$O_2max_)	HR_max_ (Beats·min^−1^)
23.1 ± 3.8	176.3 ± 6.3	70.0 ± 6.2	58.2 ± 1.9	81.9 ± 4.4	196.5 ± 8.4

$\dot{V}$O_2max_ = maximum oxygen consumption during treadmill running; VT_2_ = ventilatory threshold 2 (anaerobic); HR_max_ = maximum heart rate.

**Table 2:** Values of cardiorespiratory parameters in each training session (mean ± SD).

Variable	HRC	TS	ES (95% CI)
$\dot{V}$O_2_/BM (mL·kg^−1^·min^−1^)	18.0 ± 1.9 *	10.3 ± 1.5	4.31 (2.71–5.91)
$\dot{V}$O_2_ relative to $\dot{V}$O_2máx_ (%)	30.9 ± 3.0 *	17.7 ± 2.5	4.58 (2.91–6.25)
$\dot{V}$O_2_ relative to VO_2VT2_ (%)	37.8 ± 3.5 *	21.6 ± 2.8	4.90 (3.14–6.65)
HR (beats·min^−1^)	139.0 ± 13.2 *	100.8 ± 13.8	2.71 (1.50–3.92)
HR relative to HR_máx_ (%)	70.6 ± 7.3 *	51.4 ± 6.7	2.62 (1.43–3.82)
HR relative to HR_VT2_ (%)	76.9 ± 7.6 *	55.7 ± 6.1	2.95 (1.68–4.21)
RER	1.12 ± 0.03 *	1.05 ± 0.02	2.63 (1.43–3.83)
EC (kcal·min^−1^)	5.8 ± 1.0 *	3.5 ± 0.6	2.67 (1.47–3.88)

HRC = high-intensity resistance circuit-based training; TS = traditional strength training; ES = effect size; CI = confidence interval; $\dot{V}$O_2_/BM = oxygen consumption normalized to body mass; $\dot{V}$O_2max_/BM = maximal oxygen consumption normalized to body mass; $\dot{V}$O_2VT2_ = oxygen consumption at second ventilatory threshold; HR = heart rate; HR_max_ = maximal heart rate; HR_VT2_ = heart rate at second ventilatory threshold; RER = respiratory exchange ratio; EC = energy cost; * = significant differences, *p* ≤ 0.001.

**Table 3:** Values of cardiorespiratory parameters after each training session (mean ± SD).

Variable	HRC	TS	ES (95% CI)
EC (kcal·min^−1^)	2.5 ± 0.4 *	2.0 ± 0.3	1.35 (0.38–2.33)
EPOC (L O_2_)	5.2 ± 1.4 *	2.3 ± 0.9	2.36 (1.22–3.50)
HR (beats·min^−^^1^)	103.6 ± 9.5 *	89.4 ± 8.7	1.49 (0.50–2.48)
RER	0.92 ± 0.05	0.91 ± 0.06	0.17 (−0.70–1.05)

HRC = high-intensity resistance circuit-based training; TS = traditional strength training; ES = effect size; CI = confidence interval; EC = energy cost; EPOC = excess post-exercise oxygen consumption; HR = heart rate; RER = respiratory exchange ratio; * = significant differences, *p* ≤ 0.001.
